# Establishment of a bipedal rat model of lumbar facet joint osteoarthritis using intraarticular injection of urinary plasminogen activator

**DOI:** 10.1186/s13018-022-03339-3

**Published:** 2022-10-12

**Authors:** Di Yang, Wei Hu, Hao Li, Yin-chu Shao, Ji-chun Shan, Xu Xiong, Feng Shuang

**Affiliations:** Department of Orthopedics, The 908th Hospital of Joint Logistics Support Force of Chinese People’s Liberation Army, No.1028 Jinggangshan Street, Qingyunpu District, Nanchang, 330002 Jiangxi Province China

**Keywords:** Lumbar facet joint osteoarthritis, Urinary plasminogen activator, Bipedal rat, Low back pain, Animal model

## Abstract

**Background:**

Previous studies have demonstrated that by injecting uPA into the lumbar facet joints (LFJ) of normal rats, a rat LFJOA animal model can be successfully established. However, there is no evidence that intraarticular injection of uPA can induce or much serious osteoarthritis in bipedal rats, which biomechanics is much more similar to human than normal rats. To investigate whether intraarticular injection of urinary plasminogen activator (uPA) can induce LFJOA and low back pain symptoms in bipedal rats.

**Methods:**

An experimental study on the construction of a modified animal model of lumbar facet joints osteoarthritis (LFJOA) which biomechanics is similar to human. Sprague–Dawley rats were treated with intraarticular injection of uPA in the L5–L6 facet joints (uPA group, *n* = 15) or saline (saline group, *n* = 15). The forelimbs of both two group rats were amputated. Mechanical and thermal hyperalgesia in the ipsilateral hind paws were evaluated using von Frey hairs and a thermoalgesia instrument, respectively. Toluidine blue staining, hematoxylin–eosin staining, and immunohistochemical examination of the LFJ was performed.

**Results:**

The saline group rats have not demonstrated significant osteoarthritis in rats LFJ after surgery. The uPA group has not been induced significantly higher mechanical and thermal hyperalgesia in comparison with the saline group. But intraarticular injection of uPA in biped rats induced significantly stronger articular cartilage damage, synovitis, and proliferation of synovial cells in the LFJ. Inflammatory factors such as iNOS, IL-1β, and TNF-a were more significantly expressed in bipedal rat injected with uPA (*p* < 0.05).

**Conclusions:**

Intraarticular injection of uPA can induce LFJOA in bipedal rats, while upright posture does not induce osteoarthritis in rats LFJ in the short term.

## Background

Lumbar facet joint osteoarthritis (LFJOA) is widely thought to be a leading cause of chronic low back pain [1, 2]. To determine its pathophysiological process, animal models simulating LFJOA have been established, which can help us further understand its underlying mechanism and detect potential treatment targets. Among these animal models, intraarticular injection of chemicals into the facet joints (FJ) is one of the commonly used methods, such as collagenase [3, 4], sodium iodoacetate [5, 6], and complete Freund’s adjuvant [7, 8]. It has been confirmed that these chemical materials can induce pathohistological changes of osteoarthritis (OA) in facet joints and low back pain symptoms.

Urinary plasminogen activator (uPA) can activate plasminogen to plasmin, which can induce the activation of matrix metalloproteinase (MMP) and participate in the degradation of type 2 collagen in cartilage [9–11]. We have previously established a uPA-induced LFJOA rat model, which well-produced cartilage damage, synovitis and proliferation of synovial cells, and low back pain symptoms[12]. However, from an anatomical point of view, the biomechanical structure of the rat's spine is quite different from that of the human. Due to the differences in the biomechanical structure of the FJ, the existing LFJOA models are difficult to be used in the development of FJ surgical treatment methods (such as FJ prosthesis). It is urgent to establish LFJOA animal models that more closely approximate the biomechanics of the human spine.

To imitate the biomechanical axis of human lumbar facet joint (LFJ), Wang and his colleagues have established a modified bipedal rat model of prolonged upright posture, which can induce morphologic changes similar to human disk degeneration in rat intervertebral disks [13]. And various studies have demonstrated that bipedal rats can show much more serious degeneration of the lumbar intervertebral disks [13], cervical intervertebral disks [14], ossification of the lumbar spine [15], and calcification of the lumbar cartilage endplates [16] than normal rats. Due to that these degenerative changes in spinal are very similar to that of human, the lumbar biomechanical situation of bipedal rat demonstrates its unique mechanical advantages. In this study, we injected uPA into the L5–6 FJ of bipedal rat and compared the differences in hyperalgesia, histopathological changes in FJ and the expression levels of inflammatory factors between uPA-injected bipedal rat and saline-injected bipedal rat, in order to comprehensively evaluate whether intraarticular injection of uPA can produce behavior and pathological changes related to LFJOA in bipedal rat.

## Methods

### Animals and surgery

**Fig. 1:**
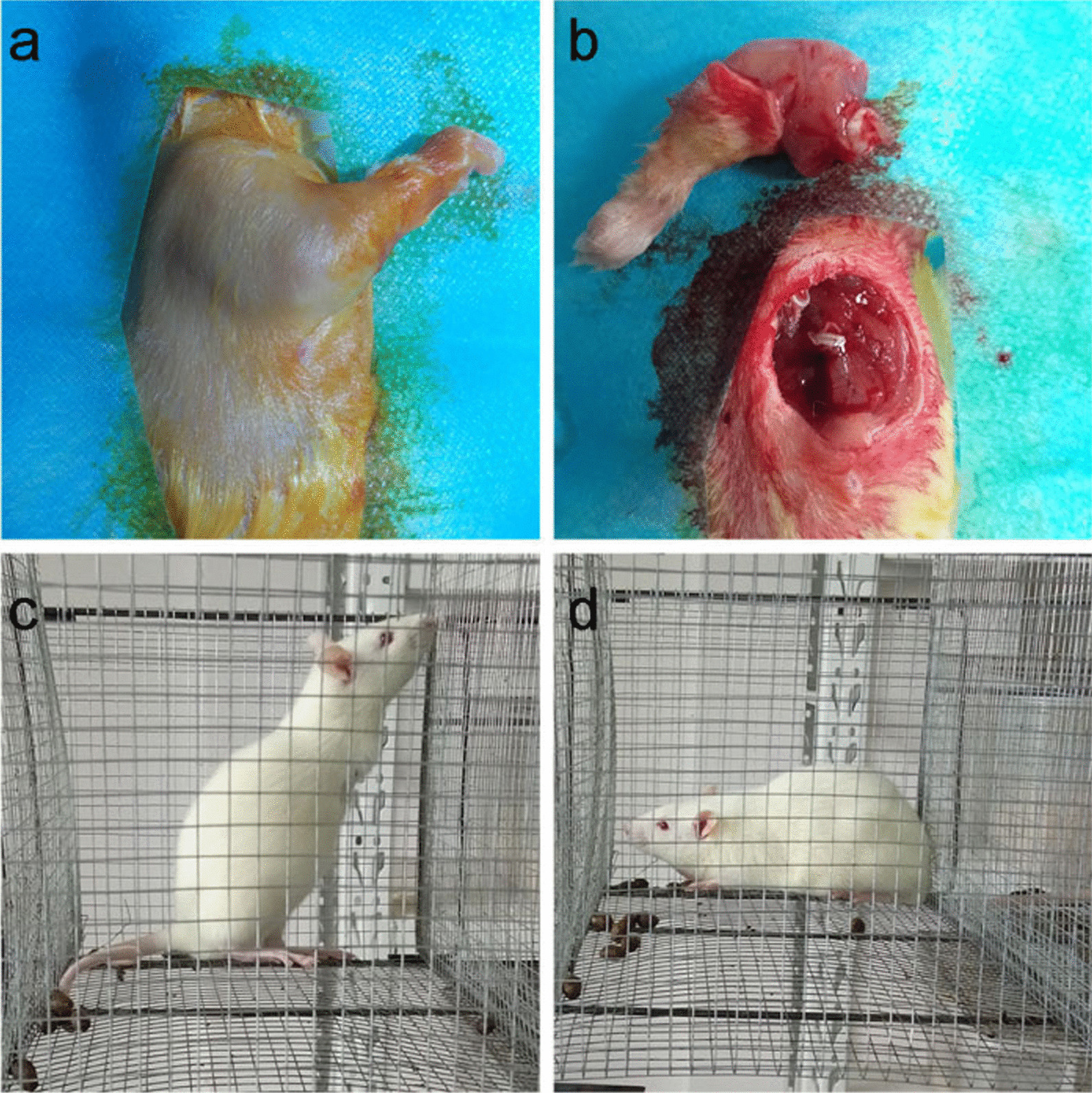
Surgery process display (**a**, **b**) and different posture before (**d**) and after (**c**) surgery

This study was approved by the Ethics Committee of the 908th Hospital of Joint Logistics Support Force of the Chinese PLA. Thirty 3-week-old male SD rats (Hunan STA Laboratory Animal Co., Ltd, China) were housed at room temperature of 22 °C with free access to food and water and 12-h light/dark cycle. All animals were randomly divided into experimental group (uPA group, *n* = 15) and control group (saline group, *n* = 15), both of which sufferer surgery. Surgical produce refers to the previous literature [12, 13]. Briefly, after general anesthesia with sodium pentobarbital (10 mg/100 g), the shoulder muscles of the rat forelimbs were incised to expose the shoulder joints, the forelimbs and scapula were separated from the joint capsule. A 1.5-cm medial incision was made on the left side of the back of the rat to expose the L5–L6 vertebral bodies, and 5ul uPA (2 mg/L, dissolved in normal saline) was injected into the joint cavity with a 34-gauge needle. The control group was injected with 5ul normal saline. Then these rats were placed on a constant temperature heating pad to maintain their body temperature and returned to the special rat cage after they woke up. The average body length of the rats in each cage was measured once a week, and the height of food and water was adjusted according to the body length to maintain the rats in an upright posture (Fig. 1).

### Mechanical hyperalgesia testing

The mechanical hyperalgesia threshold was measured before surgery and on postoperative days 3, 7, 14, 28, and 42 (*n* = 3). Rats were placed on a wire mesh with a uniform size grid, covered with a transparent plexiglass box. After the rat was acclimated to the environment for 15 min, after the rat was quiet, a standardized series of (1.0, 1.4, 2.0, 4.0, 6.0, 8.0, 10.0, 15.0, 26 g) von Frey test needles were used to vertically stimulate the rat through the wire grid. The mid-plantar of the hind limb on the operated side of the rat was bent slightly into an S shape for 6–8 s, and the paw withdrawal response was observed. The rat removed the von Frey needle within the stimulation time or when the time expired (North Coast Medical, 2007). USA), the rapid paw withdrawal response was recorded as a positive response, while the paw withdrawal response of other activities was not recorded. The test needle is gradually increased from 1.0 g, and the interval between each stimulation is 30 s. When the stimulation of the test needle cannot cause a positive response, change to a test needle with one level of strength. When testing the pressure value (g) of the needle at which 50% of the rat (measured 10 times with at least 5 consecutive paw withdrawals) withdrawal response, the paw withdrawal threshold on the side of the rat was measured.

### Thermal hyperalgesia testing

The thermal withdrawal latency of the ipsilateral hind paw was measured using a thermoalgesia instrument (BW-PL-200, Ruanlong, Shanghai) before injection and on postoperative days 3, 7, 14, 28, 42, and 56 (*n* = 3). Before test, rats were placed on a constant temperature glass plate with free movement for 15 min. Then, a thermoalgesia instrument below the glass plate was used to vertically irradiate the middle and posterior 1/3 of the hind limbs. The paw withdrawal latency refers to the ranging time(s) from irradiation to the appearance of the paw withdrawal reflex. The test procedure was repeatedly applied for 3 times with 10-min interval. To avoid thermal damage to the plantar skin, the light intensity was set to 80%, and each irradiation did not exceed 60 s.

### Cartilage toluidine blue staining

On the 7th, 14th, 28th, and 42nd days after surgery, three rats were randomly selected from two groups and sacrificed by injecting overdose of pentobarbital sodium. The L5–L6 spines were excised en bloc, fixed in 4% paraformaldehyde for 48 h, decalcified for 3 weeks, and embedded in paraffin. Then the blocks were cut into 4-µm sections on the coronal plane and stained with toluidine. Blue microscopic examination was conducted to evaluate the degree of articular cartilage degeneration according to e Osteoarthritis Research Society International (OARSI) histopathology grading system [17].

### Hematoxylin–eosin (HE) staining of the synovium

HE staining of L5–6 facet joints synovium was performed in order to evaluate synovial inflammatory condition. Synovitis scoring was based on three criteria including synovial cell proliferation, subsynovial inflammation and angiogenesis. The sum of each the terms’ scores (3-point scale) was considered as total pathology score (0–9). Synovial cell proliferation was scored as follows: 0 point for less than 3 layers, 1 point for 3–4 layers, 2 points for 5–6 layers, and 3 points for more than 6 layers. Inflammation degree was scored as follows: 0 point for no lymphocyte infiltration, 1 point for lymphocyte aggregation, 2 points for lymphoid follicles, and 3 point for lymphoid follicles with germinal center formation. Angiogenesis was scored as follows: 0 for none, 1 for mild, 2 for moderate, and 3 for severe.

### Immunohistochemial staining

The sections were incubated with primary antibodies (IL-1β (bs-0812R, Bioss, Beijing, China; 1:200); TNFa (AF7014, Affinity, Ohio; 1:200); iNOS (AF0199, Affinity, Ohio; 1:200)) at 4℃overnight. HRP-AffiniPure Goat Anti-Rabbit IgG (H + L) (BM3894, Boster, Wuhan, China; 1:1000) was incubated at 37℃for 30 min. The staining was displayed using Diaminobenzidine. Cells with only blue nuclei are negative, and cells with brownish yellow or tan granules in the cytoplasm are positive cells. The staining intensity was scored as the following: 0 point for no staining; 1 point for weak positive (light yellow staining); 2 point for moderated positive (brownish yellow); 3 point for strong positive. The positive cells in three randomly selected field were scored as the following: 0 point for negative; 1 point for less than 10%; 2 point for 11–50%; 3 point for 51–75%; 4 point for more than 75%.

### Statistical analysis

All data were expressed as mean ± standard deviation. SPSS 22.0 software (SPSS, US) was used for statistical analysis, and *t*-test was used to determine significant differences, and *p* < 0.05 was regarded as statistically significant.

## Results

### Paw mechanical and thermal withdrawal threshold

**Fig. 2:**
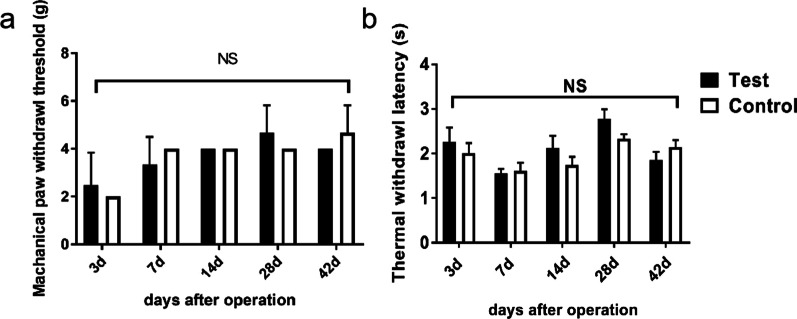
Comparison of mechanical paw withdraw threshold (**a**) and thermal withdraw latency (**b**). *: *p* < 0.05; NS: no significance

There was no significant difference in neither the paw withdrawal threshold nor the thermal withdrawal latency between the saline group rats and uPA group rats at each time point after surgery (Fig. 2, *p* > 0.05).

### Histopathologic evaluation of cartilage degeneration

**Fig. 3:**
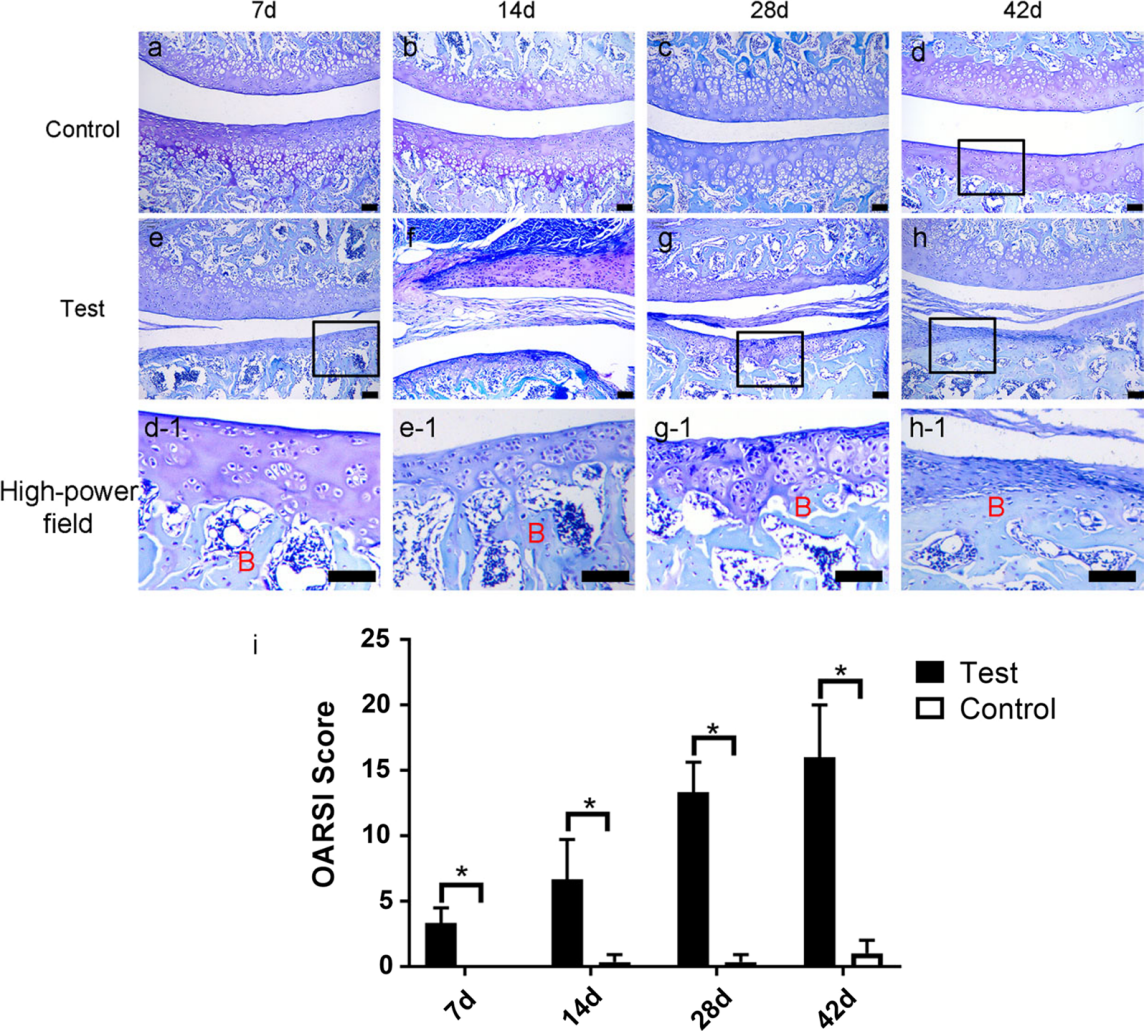
Histology of facet joint cartilage stained with toluidine blue in saline-injected control (**a**, **b**, **c** and **d**) and the uPA-injected group (**e**, **f**, **g**, and **h**) at 7, 14, 28, and 42 days after surgery. Cartilage was intact in control at 42 days after surgery (d-1). Progressive destruction of cartilage was found in uPA-injected group (e-1, g-1, and h-1). OARSI score of each group at different check points was calculated (**i**). B: bone tissue. Scale bar = 100 μm; *: *p* < 0.05; NS: no significance

As shown in Fig. 3, for saline group rats, articular cartilage surface at each time point was smooth, the structure of each layer was complete, and there was no pathological changes (Fig. 3a–d, d-1). However, for uPA group rats, their articular cartilage surface layer degenerated with disappearance of typical three-layer cartilage structure at 7 days post-surgery, chondrocytes hyperplasia and hypertrophy were also observed (Fig. 3e-1). When at D28, the joint surface of the uPA group rats was further damaged, the cartilage layered structure disorder was more obvious, and the chondrocyte hyperplasia and hypertrophy were further developed (Fig. 3g-1). At D48, the articular cartilage was basically peeled off, and the fibrous tissue directly covered the subchondral bone in the uPA group (Fig. 3h-1). The OARSI score of the uPA group was significantly higher than that of the saline group at each time point (Fig. 3.i, *p* < 0.05), which means that the uPA group had significant articular cartilage damage, and the pathological changes of cartilage OA.

### Histological evaluation of synovitis

**Fig. 4:**
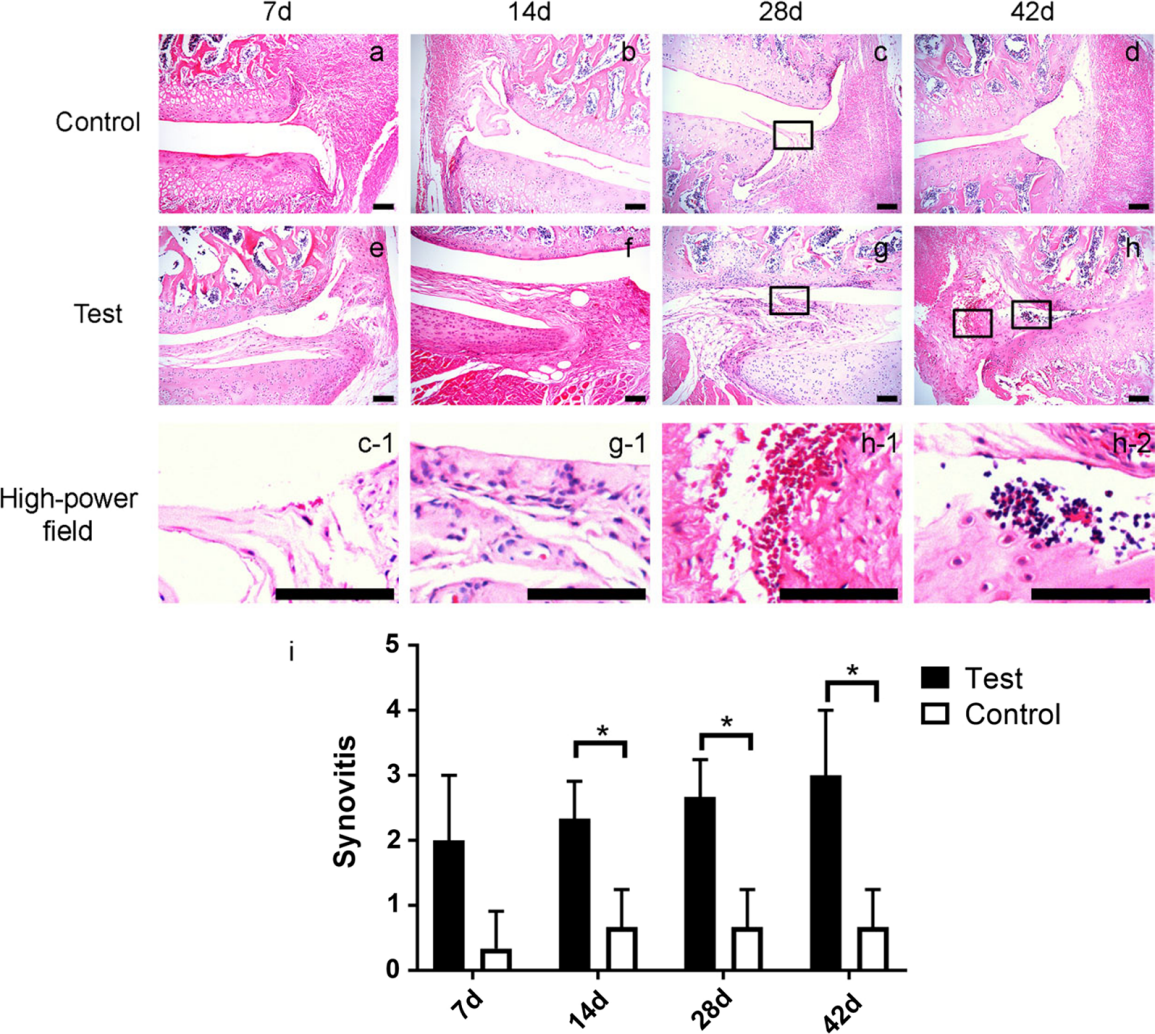
Histology of synovium stained with HE in saline-injected control (**a**, **b**, **c** and **d**) and the uPA-injected groups (**e**, **f**, **g**, and **h**) at 7, 14, 28, and 42 days after surgery. Synovium with little synovial cell proliferation, little vascularization and little inflammatory cells infiltration was found in control at 28 days after surgery(c-1). Synovial cell proliferation (g-1) was found in uPA-injected group at 28 days after surgery. Vascularization (h-1) and inflammatory cells infiltration were found in uPA-injected group at 42 days after surgery. Synovitis score of each group at different check points was calculated (**i**). Scale bar = 100 μm; *: *p* < 0.05; NS: no significance

As HE staining shown, synovitis in uPA group was significantly much serious than the saline group at each time point (Fig. 4a–h). We found that compared with the saline group, the synovial epithelial hyperplasia in the uPA group was much more obvious (Fig. 4c-1, g-1). At the same time, the vascular proliferation and inflammatory cell infiltration under the synovium in the uPA group were significantly enhanced (Fig. 4h-1, h-2). And at 14d, 28d, and 42d after operation, the synovitis score of the uPA group was significantly higher than that of the saline group (*p* < 0.05), which demonstrated that the uPA group rats had a significant inflammatory response of synovial membrane.

### IHC evaluation of arthritis factors

**Fig. 5:**
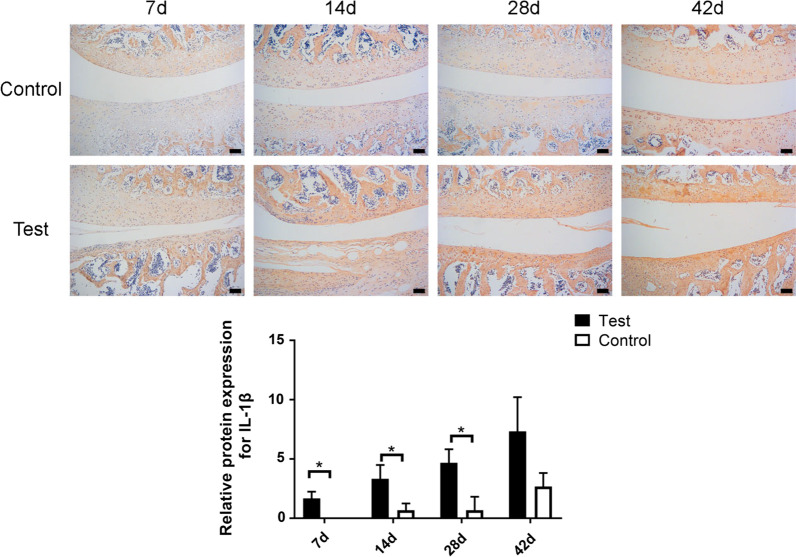
Immunohistochemical staining of IL-1β in saline-injected control and the uPA-injected groups at 7, 14, 28 and 42 days after surgery. Scale bar = 100 μm; *: *p* < 0.05; NS: no significance

**Fig. 6:**
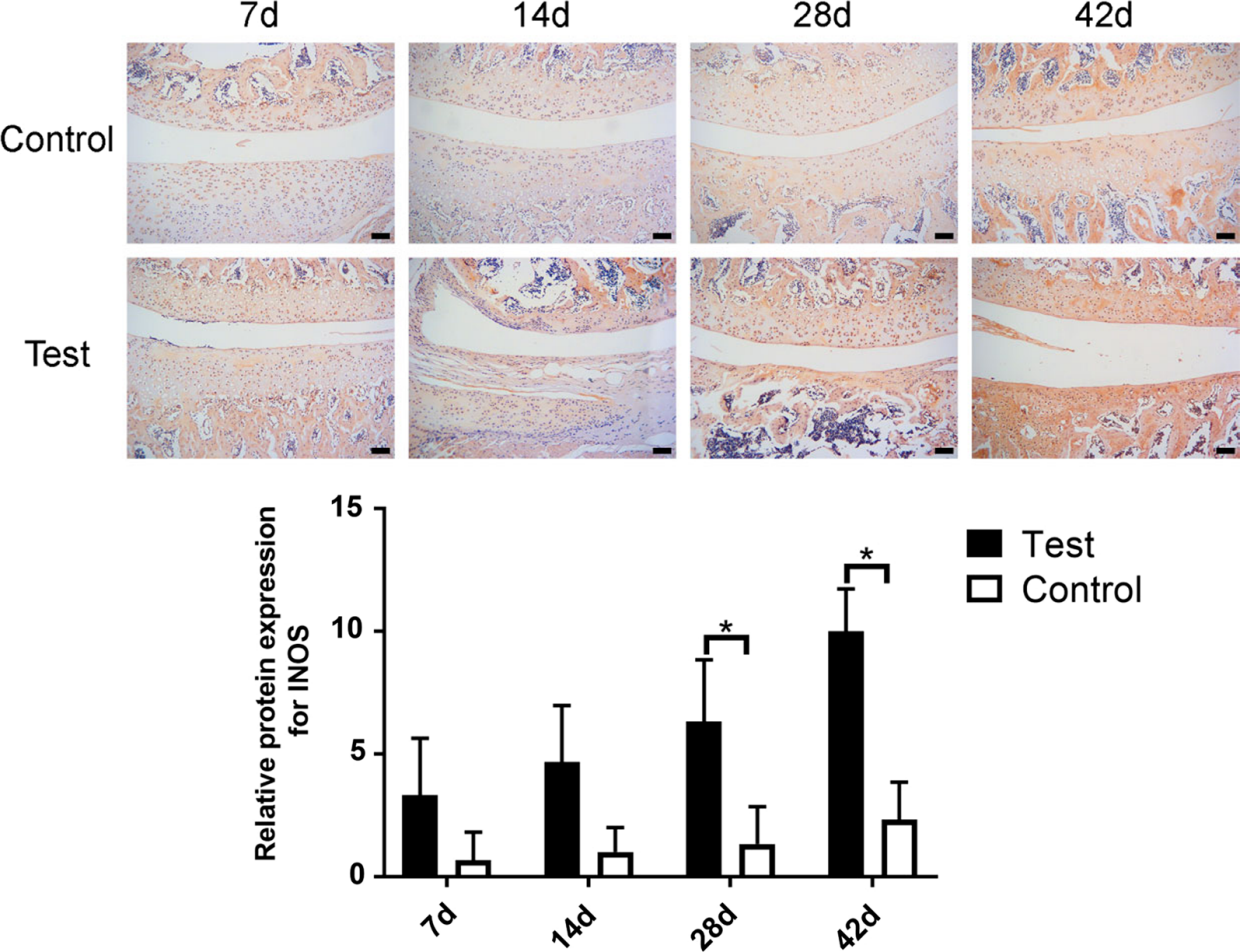
Immunohistochemical staining of iNOS in saline-injected control and the uPA-injected groups at 7, 14, 28, and 42 days after surgery. Scale bar = 100 μm; *: *p* < 0.05; NS: no significance

**Fig. 7:**
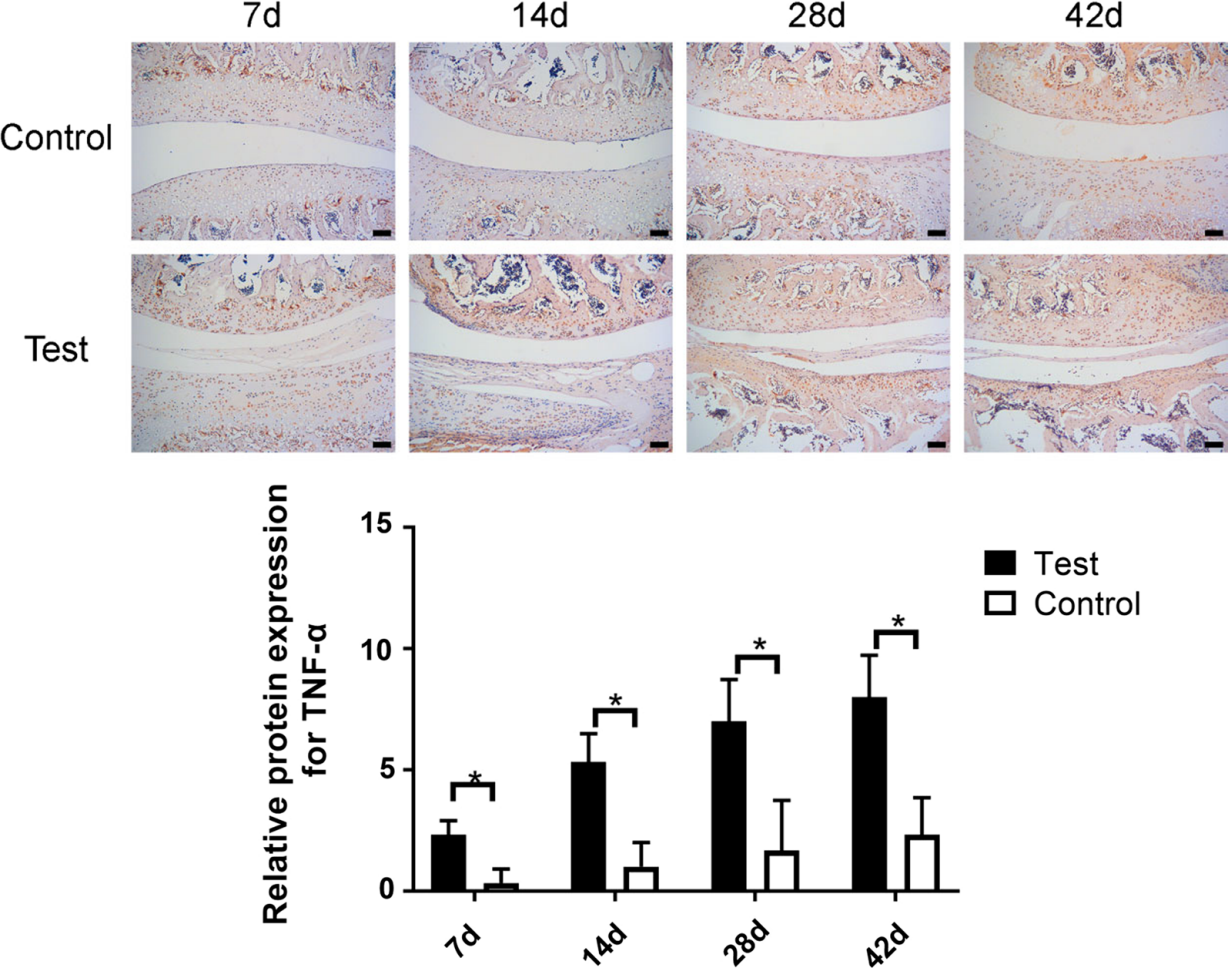
Immunohistochemical staining of TNF-α in saline-injected control and the uPA-injected groups at 7, 14, 28, and 42 days after surgery. Scale bar = 100 μm; *: *p* < 0.05; NS: no significance

As IHC staining shown, being compared with the saline group, IL-1β was more significantly highly expressed in the uPA group at 7d, 14d, and 28d after surgery, with no significant difference at 42d after surgery (Fig. 5, *p* < 0.05). The expressive level of iNOS was more significantly higher in the uPA group than the saline group at 28d and 42d after surgery, with no significant difference at short time point (7d, 14d) (Fig. 6, *p* < 0.05). And TNF-a showed significantly higher expressive level in the uPA group at each time point than the saline group (Fig. 7, *p* < 0.05).

## Discussion

Surgical treatment of LFJOA includes facet joint resection, facet joint fusion, and facet joint replacement [2]. The preclinical development of the above surgical treatment mostly depends on the establishment of animal models. Currently, there were a variety of osteoarthritis models, which can be divided into spontaneous, mechanically induced, and chemically induced model [5–8].Wang et al. [13] have established one modified bipedal rat model which biomechanics characteristics is similar to that of human spine and found that prolonged upright posture would induce degenerative changes in intervertebral disks in rat lumbar spine. In this study, we explored whether LFJOA could be induced by injecting uPA into the modified bipedal rat model, with a view to constructing a newly animal model for LFJOA. Considering that the degenerative changes of the spine in bipedal rat always occur more than 5 months after surgery [13, 14, 16], we only observed the relevant parameters in the early period after surgery.

Low back pain is a prominent symptom of LFJOA. Mechanical hyperalgesia test and thermal hyperalgesia test can more objectively reflect the degree of low back pain in animal models [18, 19]. For example, Gong et al. reported that a biphasic pattern of mechanical hyperalgesia was successfully induced in a rat LFJOA model injected with sodium iodoacetate [6]. Similarly, Hisayoshi Tachihara et al. reported that mechanical hyperalgesia was successfully induced in a rat LFJOA model implanted with complete Freund's adjuvant [7].

In this study, we found that at each time point after operation, the bipedal rat injected with uPA showed significant mechanical hyperalgesia and thermal hyperalgesia, and there was no significant difference between the two groups. Our previous study has revealed that uPA can successfully induce mechanical and thermal hyperalgesia in normal rats [12]. We speculate that amputation of both fore limbs will lead to increased sensitivity of hindlimb nerves resulting in a reduction in pain thresholds, which can mask the hyperalgesic effects due to LFJOA. It suggests that hyperalgesia could not be suitably used to evaluate the effect of LFJOA treatment in bipedal rat.

Similar to osteoarthritis of other synovial joints, LFJOA is also considered to be a degeneration of the entire joint, including destruction of articular cartilage, synovitis, etc. [20, 21]. Therefore, to evaluate whether the establishment of the LFJOA animal model is effective, the histopathological characteristics of OA are often analyzed. In this study, we focused histopathological studies on articular cartilage destruction and synovial inflammatory changes. We found that uPA group rats exhibited severe articular cartilage damage (Fig. 4), which was similar to the uPA-induced model of normal rat [12]. However, it is worth noting that injection of uPA seems to induce much serious cartilage damage in bipedal rat compared to the uPA-induced model of normal rat [12]. On the 42d after surgery, the articular cartilage in the uPA group showed extensive exfoliation, indicating that the damage to the cartilage was more serious [17]. In contrast, in the previous the uPA-induced model of normal rat, we did not find such severe articular cartilage damage at 42d after surgery. The increased degree of cartilage damage may be related to the fact that prolonged upright posture increases cartilage stress and promotes cartilage degradation [22, 23].

In addition to cartilage destruction, synovitis is also a distinguishing feature of OA [24]. Ye et al. [4] reported that injection of collagenase into the facet joints of the lumbar spine in rats induced severe synovitis. Our study found that uPA group bipedal rats exhibited serious synovitis (Fig. 5), which was similar to our previous uPA-induced model of normal rats [12]. And our study also showed that uPA-injection can induce a persistent non-resolving synovitis change ranging from d4 to d42 after surgery in the modified bipedal rat model [12], whereas at d7 post-surgery saline group did not show facet joint osteoarthritis changes. The persistent unresolved synovitis changes are more consistent with the synovial pathological changes of human LFJOA. Currently, OA has been widely accepted as an inflammatory disease [25]. The induction and progression of OA involve many inflammatory cytokines which can boost chondrocytes to release cartilage-degrading proteinases [26]. Among them, IL-1β and TNF-a play crucial roles in the catabolism of articular cartilage, promoting and maintaining the progression of OA [27]. Luo et al. found that in the rat LFJOA model injected with sodium iodoacetate, the levels of IL-1β and TNF-a in synovial fluid increased. IL-1β and TNF-a can stimulate chondrocytes and synovial cells to produce iNOS, which in turn produces NO, which promotes cartilage apoptosis [28]. In this study, we also found that the joint cartilage of the uPA group rat expressed much higher IL-1β, TNF-a and iNOS [12]. Similarly, our study revealed that intraarticular injection of uPA in bipedal lumbar facet joints induced facet joint osteoarthritis changes, whereas upright posture did not induce facet joint osteoarthritis changes in rats in the short term.

## Conclusions

In conclusion, we firstly reported that facet joint osteoarthritis can be induced in bipedal rats by intraarticular injection of uPA, whereas upright posture alone does not induce facet joint osteoarthritis in the short term. This model incorporates the biomechanical properties of the human spine into the consideration of LFJOA modeling, which can be used to develop preclinical studies targeting surgical treatment of LFJOA.

## Data Availability

All data generated or analyzed during this study are included in this published article.
